# Immune escape of multiple myeloma cells results from low miR29b and the ensuing epigenetic silencing of proteasome genes

**DOI:** 10.1186/s40364-024-00592-y

**Published:** 2024-04-23

**Authors:** Patrizia Leone, Eleonora Malerba, Marcella Prete, Antonio Giovanni Solimando, Giorgio Alberto Croci, Paolo Ditonno, Marco Tucci, Nicola Susca, Afshin Derakhshani, Antoine Dufour, Valli De Re, Nicola Silvestris, Vito Racanelli

**Affiliations:** 1 Department of Interdisciplinary Medicine, Aldo Moro University of Bari, Bari, Italy; 2 Department of Precision and Regenerative Medicine and Ionian Area-(DiMePRe-J), Aldo Moro University of Bari, Bari, Italy; 3 Division of Pathology, Foundation IRCCS Ca’ Granda Ospedale Maggiore Policlinico, Milan, Italy; 4 Hematology Unit, IRCCS “Giovanni Paolo II”, Bari, Italy; 5 Department of Microbiology, Immunology, and Infectious Diseases, Calvin, Phoebe and Joan Snyder Institute for Chronic Diseases, Cumming School of Medicine, University of Calgary, Calgary, AB Canada; 6 McCaig Institute for Bone and Joint Health, University of Calgary, Calgary, Canada; 7 Department of Microbiology, Immunology, and Infectious Diseases, Cumming School of Medicine, University of Calgary, Calgary, Canada; 8 Department of Physiology and Pharmacology, University of Calgary, Calgary, AB Canada; 9 Bio-Proteomics Facility, Department of Translational Research, Centro Di Riferimento Oncologico Di Aviano (CRO) IRCCS, Aviano, PN Italy; 10 Medical Oncology Unit, Department of Human Pathology “G. Barresi”, University of Messina, Messina, Italy; 11 Centre for Medical Sciences, University of Trento and Internal Medicine Division, Santa Chiara Hospital, Provincial Health Care Agency (APSS), Trento, Italy

**Keywords:** Multiple myeloma, MGUS, Proteasome, T cells, Plasma cells, Tumor immune evasion, Bone marrow

## Abstract

**Background:**

Activation of CD28 on multiple myeloma (MM) plasma cells, by binding to CD80 and CD86 on dendritic cells, decreases proteasome subunit expression in the tumor cells and thereby helps them evade being killed by CD8^+^ T cells. Understanding how CD28 activation leads to proteasome subunit downregulation is needed to design new MM therapies.

**Methods:**

This study investigates the molecular pathway downstream of CD28 activation, using an in vitro model consisting of myeloma cell lines stimulated with anti-CD28-coated beads.

**Results:**

We show that CD28 engagement on U266 and RPMI 8226 cells activates the PI3K/AKT pathway, reduces miR29b expression, increases the expression of DNA methyltransferase 3B (DNMT3B, a target of miR29b), and decreases immunoproteasome subunit expression. In vitro transfection of U266 and RPMI 8226 cells with a miR29b mimic downregulates the PI3K/AKT pathway and DNMT3B expression, restores proteasome subunit levels, and promotes myeloma cell killing by bone marrow CD8^+^ T cells from MM patients. Freshly purified bone marrow plasma cells (CD138^+^) from MM patients have lower miR29b and higher DNMT3B (mRNA and protein) than do cells from patients with monoclonal gammopathy of undetermined significance. Finally, in MM patients, high *DNMT3B* levels associate with shorter overall survival.

**Conclusions:**

Altogether, this study describes a novel molecular pathway in MM. This pathway starts from CD28 expressed on tumor plasma cells and, through the PI3K-miR29b-DNMT3B axis, leads to epigenetic silencing of immunoproteasome subunits, allowing MM plasma cells to elude immunosurveillance. This discovery has implications for the design of innovative miR29b-based therapies for MM.

**Supplementary Information:**

The online version contains supplementary material available at 10.1186/s40364-024-00592-y.

## Introduction

Multiple myeloma (MM) is a plasma cell malignancy characterized by the growth of tumor cells in the bone marrow. MM is often preceded by an asymptomatic premalignant condition termed monoclonal gammopathy of undetermined significance (MGUS) [1, 2] that progresses to MM at a rate of approximately 1% per year [3]. We previously established that MGUS-to-MM progression is characterized by plasma cell downregulation of several genes encoding proteasome subunits (δ, MB1, Z, LMP2, LMP7, LMP10) that are involved in tumor antigen processing [4]. This event reduces the generation of human leukocyte antigen (HLA) class I-restricted peptides, which are normally presented to tumor-specific CD8^+^ T cells, and ultimately leads to plasma cell evasion of immune recognition and killing.

Our research has also revealed that the downregulation of proteasome subunit genes occurs when CD28 on MM plasma cells binds its ligands CD80 and CD86 on dendritic cells [5]. However, the molecular events that link CD28 activation to proteasome subunit downregulation have not been determined. Some clues suggest that they involve epigenetic gene silencing through DNA methylation. For instance, CD28 activation on MM plasma cells triggers phosphatidylinositol-3-kinase (PI3K)/AKT signaling [6, 7], and PI3K/AKT signaling supports aberrant DNA methylation in several tumors [8, 9]. Furthermore, treatment of MM plasma cells with decitabine, an inhibitor of DNA methyltransferases (DNMTs), raises the expression of proteasome subunits to levels comparable to those in MGUS plasma cells [4]. Interestingly, the microRNA miR29b, a member of the miR29 family, modulates the methylation profile of MM plasma cells by targeting mainly *DNMT3B* [10]. Downregulation of miR29b is characteristic of MM cell lines and primary MM plasma cells [11]. Through imperfect base-pairing, miR29b binds the 3’ untranslated region of *DNMT3B* mRNA, destabilizes it and inhibits its translational, with a subsequent reduction of overall DNA methylation [12]. Given that abnormal miR29b expression patterns have been reported in various tumor cells (reviewed in [13]), miR29b is considered to be an epigenetic controller (epi-miRNA) that is involved in tumor development and progression [13].

In this study, we tried to recapitulate in vitro what occurs in vivo when MM plasma cells interact with dendritic cells in the bone marrow microenvironment. We tested whether the stimulation of CD28 activates the PI3K/AKT pathway in MM plasma cells and whether this activation influences the expression of miR29b and the activity of DNMT3B. We also analyzed the effect of this stimulation on the methylation status as well as the expression of proteasome subunit genes and on plasma cell recognition by T cells.

## Methods

### Cell culture

The human U266 and RPMI 8226 myeloma cell lines (U266B1, CVCL_0566; RPMI-8226, CVCL_0014) were obtained from the American Type Culture Collection (ATCC) and cultured in RPMI-1640 medium supplemented with 10% fetal bovine serum (FBS), 2 mM L-glutamine, 100 U/mL penicillin, and 100 μg/mL streptomycin (all from Sigma-Aldrich). Cells were grown at 37 °C in a humidified atmosphere containing 5% CO_2_.

### Bone marrow sampling and cell isolation procedures

Bone marrow was obtained from consecutive patients who were newly diagnosed in 2019–2022 with a monoclonal gammopathy or a benign hematological disorder (considered “normal” controls). Patients were classified as having MGUS (*n* = 20) or symptomatic MM (*n* = 25) according to the International Myeloma Working Group criteria [14] or benign hematological disorders (*n* = 10) (Table 1). Bone marrow was sampled by both aspiration and biopsy of the posterior iliac crest. Bone marrow samples were subjected to HLA molecular typing, and subgroups of MM patients that were HLA-A*0201–negative or HLA-A*0201–positive were used in certain experiments.

**Table 1:** Clinical characteristics of patients

Characteristic	Controls	MGUS	MM
No. patients	10	20	25
Median age, years (range)	65 (52—77)	62 (49—75)	66 (47—81)
Sex			
Male, n (%)	6 (60)	12 (60)	13 (52)
Female, n (%)	4 (40)	8 (40)	12 (48)
Iron deficiency anemia, n (%)	7 (70)	N.A	N.A
Thrombocytopenia, n (%)	3 (30)	N.A	N.A
Immunoglobulin subtype, n (%)			
IgG k	N.A	16 (80)	18 (72)
IgG λ	N.A	2 (10)	3 (12)
IgA k	N.A	1 (5)	3 (12)
IgA λ	N.A	1 (5)	1 (4)
International Staging System, n (%)			
I	N.A	N.A	4 (16)
II	N.A	N.A	10 (40)
III	N.A	N.A	11 (44)

*Abbreviations*: *MGUS* monoclonal gammopathy of undetermined significance, *MM* multiple myeloma, *Ig* immunoglobulin, *N.A.* not applicable

Bone marrow mononuclear cells (BMMC) were isolated by density gradient centrifugation on Ficoll-Paque Plus (GE Healthcare Life Sciences). Then, plasma cells (CD138^+^) were purified from BMMC by automated magnetic sorting using anti-CD138 microbeads (Miltenyi Biotec). The sorted cells exhibited > 95% purity, as revealed by flow cytometry on immunostained cells. CD138^+^ sorted cells were frozen as dry pellets and stored at -80 °C until further use.

BMMC were also used to generate epitope-specific CD8^+^ T cells for cytotoxicity assays. For this purpose, we used BMMC from the five HLA-A*0201–positive MM patients, given that the target cells for cytotoxicity assays were U266 cells that express HLA-A*0201 (Cellosaurus CVCL_0566). We cultured BMMC (5 × 10^5^/well) in 96-well round-bottom plates (BD Biosciences) in 200 µL/well TexMACS medium (Miltenyi Biotec) containing 10% FBS, 100 U/mL penicillin, and 100 μg/mL streptomycin. Starting at seeding, cells were stimulated with 10 µg/mL NY-ESO-1_157–165_ (SLLMWITQV) peptide (Proimmune) in culture medium. This HLA-A*0201-restricted peptide is derived from cancer/testis antigen 1B (alias NY-ESO-1), which is highly expressed by U266 cells [4]. The culture medium was replaced with fresh medium containing 10 U/mL rIL-2 (PeproTech) on days 4, 7, 11, 14 and 18; on day 7, the fresh medium also contained 10 ng/mL rIL-7 (PeproTech). Cultures were restimulated with irradiated autologous BMMC (5 × 10^5^/well) plus peptide on days 7 and 14. On day 21, cells were harvested, pooled and used for magnetic CD8^+^ T cell isolation using anti-CD8 microbeads (Miltenyi Biotec). Antigen specificity of the generated T cells was evaluated by NY-ESO-1_157–165_ pentamer staining and flow cytometry. The cells were used as effectors in cytotoxicity assays.

### Cell treatments

U266 and RPMI 8226 cells (4 × 10^6^ cells) were treated in complete culture medium with 10 µg/mL anti-CD28-coated beads (Miltenyi Biotec) without or with 50 µg/mL mouse anti-human CD28-blocking monoclonal antibody (clone CD28.6, eBioscience, Thermo Fisher Scientific) for 24 h. In other assays, cells were treated with 25 µM LY294002 for 48 h or 0.5 µM buparlisib for 24 h (both PI3K inhibitors; Selleckchem Aurogene, Rome, Italy), or 1 µM 5-aza-2′-deoxycytidine (decitabine, Sigma-Aldrich) for 72 h. Treated cells were used in western blotting and real-time PCR.

### Western blotting

Treated cells (3 × 10^6^ per sample) were washed and lysed in RIPA lysis buffer (#20–188) supplemented with protease inhibitors (#P8340) (both from Sigma-Aldrich). Lysates were quantified for protein using the Bradford assay (Bio-Rad), and 35 µg per sample was used in western blotting using the monoclonal antibodies (mAb) given in Supplemental Table 1. Bound primary antibodies were detected with horseradish peroxidase-conjugated goat anti-mouse IgG (#170–6516, 1:3000) or goat anti-rabbit IgG (#170–6515, 1:3000) and visualized by enhanced chemiluminescence using Clarity Western ECL Blotting Substrate with a ChemiDoc XRS + imaging system (all from Bio-Rad). Bands were quantified as optical density units using Image Lab software (Bio-Rad). Results were expressed as relative density normalized to the control condition.

### Real-time PCR

Total RNA and miRNA were extracted from U266 and RPMI 8226 cells or immunomagnetically purified plasma cells using the mirVana miRNA Isolation Kit (Thermo Fisher Scientific, #AM1560) according to the manufacturer’s instructions. Total RNA concentration and purity ranged 54–135 ng/µl and 1.98–2.01 (260/280 OD ratio), respectively. miRNA concentration and purity ranged 3–10 ng/µl and 1.96–2.08 (260/280 OD ratio), respectively. cDNA was synthesized from total RNA using SuperScript IV VILO Master Mix (Thermo Fisher Scientific, #11766050) according to the manufacturer’s instructions. miRNA was reverse-transcribed using the TaqMan MicroRNA Reverse Transcription Kit (Thermo Fisher Scientific, #4366596) and miRNA-specific stem-loop primers for miR29b (#RT000413) and RNU44 (internal control, #RT001094). The thermal cycler was set up as recommended in the kit protocol. Levels of mRNA for *DNMT3B* and glyceraldehyde-3-phosphate dehydrogenase (*GAPDH*) and of miR29b and RNU44 were determined in triplicate by real-time PCR using TaqMan kits (Thermo Fisher Scientific assay IDs: Hs00171876_m1, Hs99999905_m1, hsa-miR-29b 000413, RNU44 001094).

Real time PCR was performed in 20µl reaction mixture that included 2X TaqMan Universal Master Mix II (#4440038), 20X TaqMan assay, nuclease free water (all from Applied Biosystems) and 20 ng cDNA. Amplification and fluorescence readings were performed on a StepOnePlus Real-Time PCR System (Thermo Fisher Scientific) at 50 °C for 2 min (uracil DNA glycosylase incubation), 95 °C for 10 min (polymerase activation), 45 cycles of 95 °C for 15 s (denaturation) and 60 °C for 1 min (annealing/extension). mRNA levels were normalized to that of GAPDH for each sample and then expressed as fold change relative to the average value from controls. miR29b levels were normalized to that of RNU44 for each sample and then expressed as fold change relative to the average value from controls. Analyses were performed with StepOne Software (Thermo Fisher Scientific) using the 2^−ΔΔCt^ method. GAPDH was chosen over β2m and TBP because it resulted to be the most stable housekeeping gene under our experimental conditions, according to the NormFinder algorithm [15].

### Transient transfection with a miR29b mimic

U266 and RPMI 8226 cells were seeded in 24-well round-bottom plates (BD Biosciences) at a density of 3 × 10^5^ cells/well and immediately transfected with 10 µM synthetic miR29b mimic (miR29b, Thermo Fisher Scientific #4464066) or miRNA mimic negative control (miR-NC, Thermo Fisher Scientific #4464058) using Lipofectamine RNAiMAX (Thermo Fisher Scientific, #13778030). Transfected cells were cultured in 500 µL medium per well for 72 h before being harvested and used for flow cytometry or as targets in cytotoxicity assays, or processed for miRNA, RNA or protein. Cell death after transfection was < 25% as revealed by trypan blue staining.

### Flow cytometry

Cells were stained for surface and intracellular proteins as described previously [5] using fluorochrome-conjugated mAb from both commercial and non-commercial sources (Supplemental Table 1) or PE-conjugated NY-ESO-1_157–165_–specific HLA-A*0201 pentamer (Proimmune, Oxford, UK). The non-commercial mAb (provided by S. Ferrone; Massachusetts General Hospital, Harvard Medical School, Boston, USA) whose production and characterization have already been reported [16], were used as described previously [5].

For surface staining, cells were incubated with mAb to surface antigens for 30 min at 4 °C and then washed twice in cold phosphate-buffered saline containing 0.1% bovine serum albumin before flow cytometry. Staining with pentamers was performed according to Proimmune’s instructions. In some cases, surface-stained cells were fixed and permeabilized with BD Cytofix/Cytoperm solution (BD Biosciences), washed in Perm/Wash solution (BD Biosciences), and stained with mAb to intracellular proteins at 4 °C for 30 min.

For tumor plasma cell (CD138^+^) enumeration, whole bone marrow samples were incubated with an anti-CD138 mAb and mixed with Flow-Count Fluorospheres (Beckman Coulter) at known concentrations, as previously described [5], immediately before flow cytometry. Values were expressed as cells per microliter.

Stained cells were analyzed without delay on a FC500 flow cytometer (Beckman Coulter) using CXP software (Beckman Coulter) and FlowJo software. For CD8^+^ T cells stained with pentamers, the level of nonspecific binding was calculated from the background signal observed in cells from five HLA-A*0201–negative MM patients. In particular, the cutoff for a pentamer-positive signal was set as the average background signal plus 3 SD.

### Cytotoxicity assays

Target cells for cytotoxicity assays were U266 cells transfected or treated as described earlier. Effector cells were NY-ESO-1_157–165_-specific CD8^+^ T cells purified from BMMC of five HLA-A*0201–positive MM patients, as described earlier. Target and effector cells were incubated together in round-bottom 96-well plates in TexMACS medium at 37 °C in a humidified atmosphere containing 5% CO_2_. Each well contained 2 × 10^4^ target cells and a serial dilution of effectors (from 1 × 10^4^ to 20 × 10^4^), for effector/target ratios of 0.5:1, 5:1 and 10:1. The assay was performed in triplicate. Wells containing target cells only or effector cells only served as control. After 4 h at 37 °C, specific cytotoxicity activity was measured using a colorimetric lactate dehydrogenase (LDH) assay (CytoTox 96 Non-Radioactive Cytotoxicity Assay; Promega, # G1780). Released LDH was measured according to the manufacturer’s protocol. The percentage of specific killing was calculated with the following formula: % specific killing = (experimental release − effector spontaneous release – target spontaneous release)/(target maximum release – target spontaneous release) × 100. Spontaneous LDH release was measured by incubating cells in the medium alone. Maximum LDH release was achieved by adding 0.8% Triton X-100 (Lysis solution) to target cells.

### Immunohistochemistry and in situ hybridization

Bone marrow tissue was EDTA-decalcified, formalin-fixed, and processed into 4 μm sections. Staining with an anti-DNMT3B antibody (Abcam ab2851; 1:250 dilution) was done using an automated stainer (BenchMark Ultra, Ventana-Roche Diagnostics). miR29b was detected in situ using the miRNAscope HD Assay Red (Advanced Cell Diagnostics, Newark (CA), USA) and an miR29b-specific probe (SR-hsa-miR-29b-3p-S1 MIMAT0000100, Advanced Cell Diagnostics). The negative control was a non-specific miRNA probe (Advanced Cell Diagnostics). Slides were digitalized and analyzed on an Aperio GT 450 DX slide scanner (Leica) at 400 × magnification.

### DNA methylation analysis

DNA methylation analysis was performed using Infinium MethylationEPIC microarrays which cover over 850,000 CpGs (Infinium MethylationEPIC BeadChip Kit, #WG-317–1001). Briefly, DNA was extracted from U266 cells (3 × 10^6^ per sample) and from plasma cells of MGUS (from 1 × 10^6^ to 3 × 10^6^) and MM (from 3.8 × 10^6^ to 7 × 10^6^) patients using the PureLink Genomic DNA purification kit (Thermo Fisher Scientific, # K182001). Cytosines (but not methylcytosines) in DNA (500 ng per sample) were deaminated by sodium bisulfite treatment using the EZ-96 DNA Methylation Kit (Zymo Research, #D5001). The DNA samples were denatured, amplified, enzymatically fragmented, and hybridized to BeadChips according to the Infinium HD Assay Methylation Protocol Guide (#15019519 v06). BeadChips were washed to remove unhybridized and non-specifically hybridized DNA, subjected to allele-specific single-base extension and staining, and imaged on an Illumina iScan System.

iScan data files were subjected to quality control using GenomeStudio software (version 2011.1), and the resulting files were analyzed using the Bioconductor R package Chip Analysis Methylation Pipeline (ChAMP). The methylation intensity at each CpG site was expressed as a β value that ranged from 0 (unmethylated) to 1 (methylated) [17]. We filtered out probes with *P* > 0.01, SNP-related probes, multi-hit probes, probes for the X and Y chromosomes, probes with < 3 beads in at least 5% of samples, and non-CpG probes. Signals for the remaining probes were normalized using the beta-mixture quantile method [17]. Differentially methylated CpG sites were identified by a Benjamini–Hochberg adjusted *P* < 0.05, with no threshold for the methylation difference. Differentially methylated regions were identified using the Bumphunter method [18] and defined as 300 bp regions marked by multiple probes and with an overall methylation difference between MGUS and MM patients. Three promoter regions were then analyzed: TSS1500 (200–1500 bp upstream of the transcription start site), TSS200 (0–200 bp upstream of the transcription start site) and 5’UTR (untranslated region). The methylation level of each of these regions was expressed as the average β value for the differentially methylated probes that map to it.

### Survival analysis

The impact of *DNMT3B* expression levels in MM plasma cells on overall survival of MM patients was investigated using publicly deposited gene expression and clinical data. We obtained data for 414 newly diagnosed MM patients (GSE4581) and 264 relapsed/refractory MM patients (GSE9782). Probe sets with potential prognostic significance were identified by applying the Maxstat function along with Benjamini–Hochberg multiple testing correction to the expression data, as previously described [19]. This analysis identified the best cutoff for dichotomizing patients in each cohort into high and low *DNMT3B* expression groups. Kaplan–Meier curves were generated to compare overall survival between the high and low *DNMT3B* groups.

### Statistical analysis

Nonparametric statistical tests were used because much of the data were not distributed normally. Tests included the Mann–Whitney U test for comparisons of groups, the Wilcoxon signed-rank test for comparisons of matched samples, Spearman’s rank test for correlations, and log-rank test for survival analysis. Statistical analyses were done using Prism (GraphPad Software). A value of *P* < 0.05 was taken to indicate statistical significance.

## Results

### CD28 engagement triggers the PI3K/AKT signaling pathway and regulates miR29b and DNMT3B expression

**Fig. 1:**
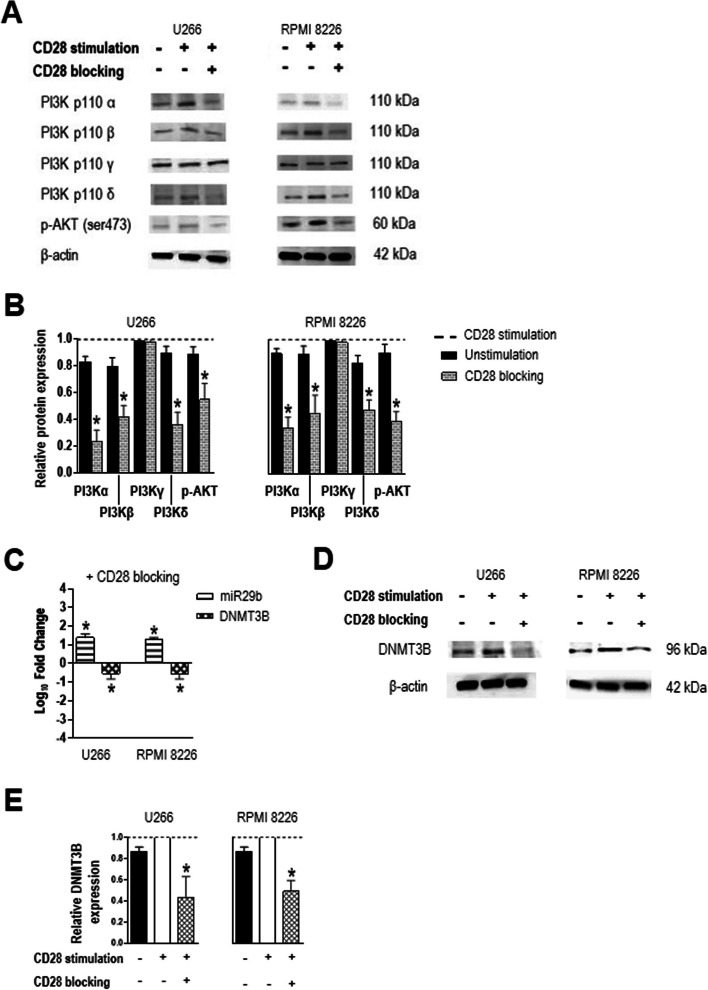
CD28 activation of the PI3K/AKT signaling pathway and regulation of miR29b and DNMT3B expression in MM plasma cells (U266 and RPMI 8226 cells). Cells were cultured in the presence of 10 µg/mL anti-CD28-loaded particles, alone or with either 50 µg/mL CD28-blocking mAb (24 h). **A** Representative western blots of PI3K/AKT pathway components in conditions of CD28 stimulation or blocking. **B** Densitometric analysis of the experiment shown in (**A**). Band intensities were normalized to those of β-actin and the relative levels of proteins under CD28 blocking were expressed as a percentage of those during CD28 stimulation (dotted line). **C** Levels of miR29b and *DNMT3B* mRNA under conditions of CD28 blocking normalized to those of RNU44 or *GAPDH*, respectively, and then expressed as fold change relative to the average value for CD28-stimulated cells (data from real-time PCR and 2^−ΔΔCt^ method). **D** Representative western blot and **E** densitometric analysis of DNMT3B expression after CD28 stimulation or blocking. Values were normalized to β-actin and then to values for CD28-stimulated cells (dotted line). Chart data are mean and SD for 7 independent experiments. *P < .05, Wilcoxon signed-rank test

To assess whether CD28 binding to its ligands CD80 and CD86 triggers PI3K signaling, we cultured U266 and RPMI 8226 myeloma cells (which constitutively express CD28, Supplemental Fig. 1) with anti-CD28-coated beads (to mimic stimulation by ligands expressed on antigen-presenting cells), in the absence or presence of a CD28-blocking mAb, and used Western blotting to examine the expression of components of the PI3K/AKT signaling pathway (Fig. 1A, B). Under conditions of CD28 stimulation, all five examined components (PI3Kα, PI3Kβ, PI3Kγ, PI3Kδ, phospho-AKT) were detected. When CD28 blockade was added, lower levels were detected for all but PI3Kγ. These results suggest that CD28 stimulation specifically increases levels of the catalytic subunits PI3Kα, PI3Kβ and PI3Kδ (for all, *P* = 0.0156) and activates the PI3K/AKT pathway, as indicated by the level of phospho-AKT that was significantly lower with CD28 blockade (*P* = 0.0156).

We used the same assay to assess whether CD28 engagement influences levels of miR29b and its target *DNMT3B* in U266 and RPMI 8226 myeloma cells (Fig. 1C-E). When CD28-stimulated cells were subjected to CD28 blockade, miR29b levels increased (*P* = 0.0156) and *DNMT3B* mRNA levels decreased (*P* = 0.0156), as did DNMT3B protein levels (*P* = 0.0156).

**Fig. 2:**
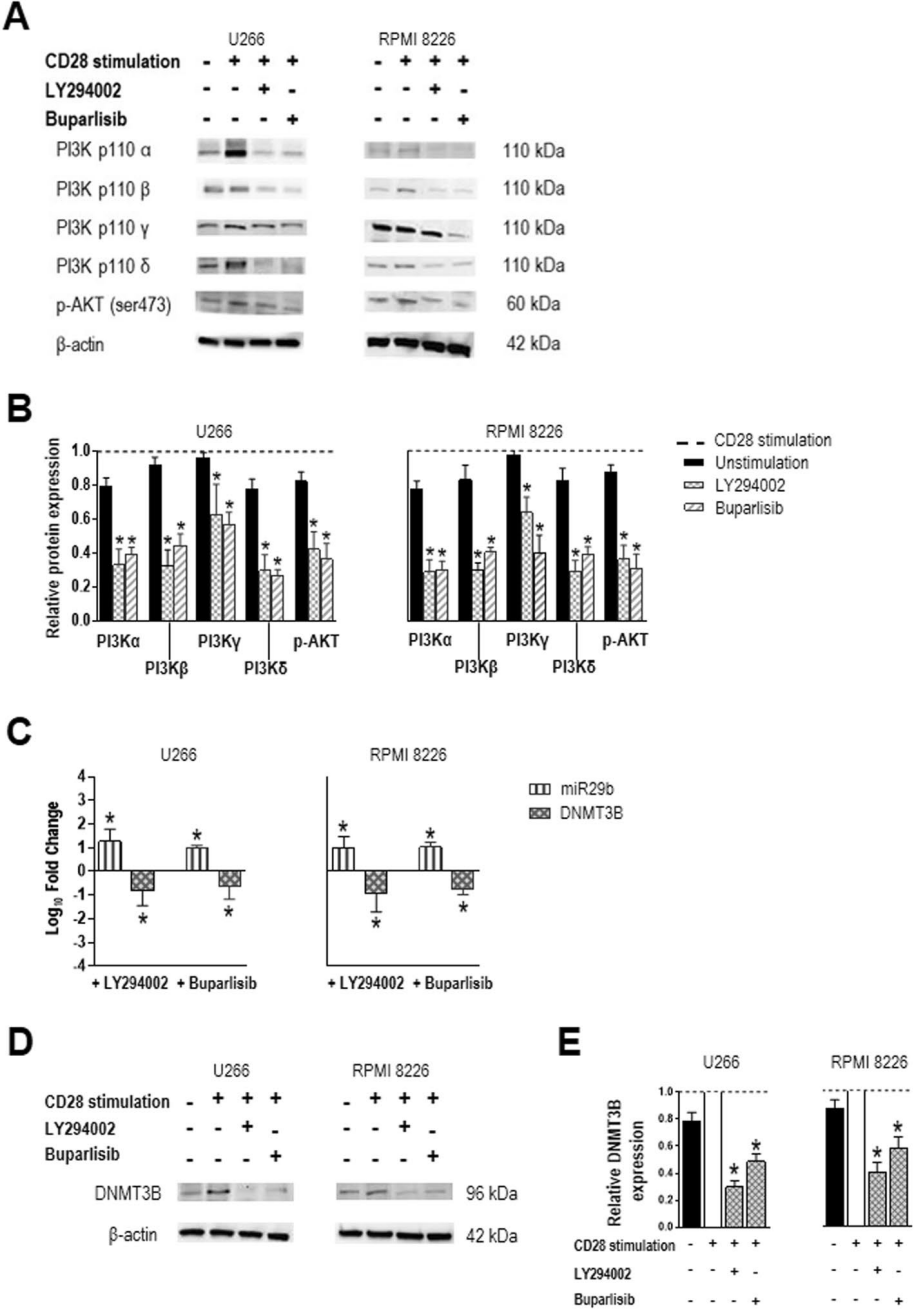
PI3K/AKT signaling pathway inhibition and regulation of miR29b and DNMT3B expression in MM plasma cells (U266 and RPMI 8226 cells). Cells were cultured in the presence of 10 µg/mL anti-CD28-loaded particles, alone or with PI3K inhibitor (25 µM LY294002 48 h; 0.5 µM buparlisib 24 h). **A** Representative western blots of PI3K/AKT pathway components under CD28 stimulation alone or with PI3K inhibition. **B** Densitometric analysis of the experiment shown in (**A**). Values were normalized to β-actin and then to values for CD28-stimulated cells (dotted line). **C** Plasma cell levels of miR29b and *DNMT3B* after PI3K inhibition normalized to those of RNU44 or *GAPDH*, respectively, and expressed as fold change relative to the average value for CD28-stimulated cells. **D** Representative western blot and **E** densitometric analysis of DNMT3B expression after CD28 stimulation and PI3K inhibition. Chart data are mean and SD for 7 independent experiments. *P < .05, Wilcoxon signed-rank test

To determine whether the regulation of miR29b and *DNMT3B* levels by CD28 involved activation of the PI3K/AKT pathway, we simultaneously exposed U266 and RPMI 8226 myeloma cells to CD28 stimulation and PI3K inhibition with the PI3K inhibitors LY294002 and buparlisib (Fig. 2). The combined treatment reduced protein levels of all examined components of the PI3K/AKT pathway compared to CD28 stimulation alone (Fig. 2A, B). Furthermore, it increased levels of miR29b and reduced levels of both *DNMT3B* mRNA (Fig. 2C) and protein (Fig. 2D, E). These results indirectly suggest that, under conditions of CD28 engagement and consequential PI3K/AKT pathway activation, miR29b levels are low and those of DNMT3B are subsequently high.

**Fig. 3:**
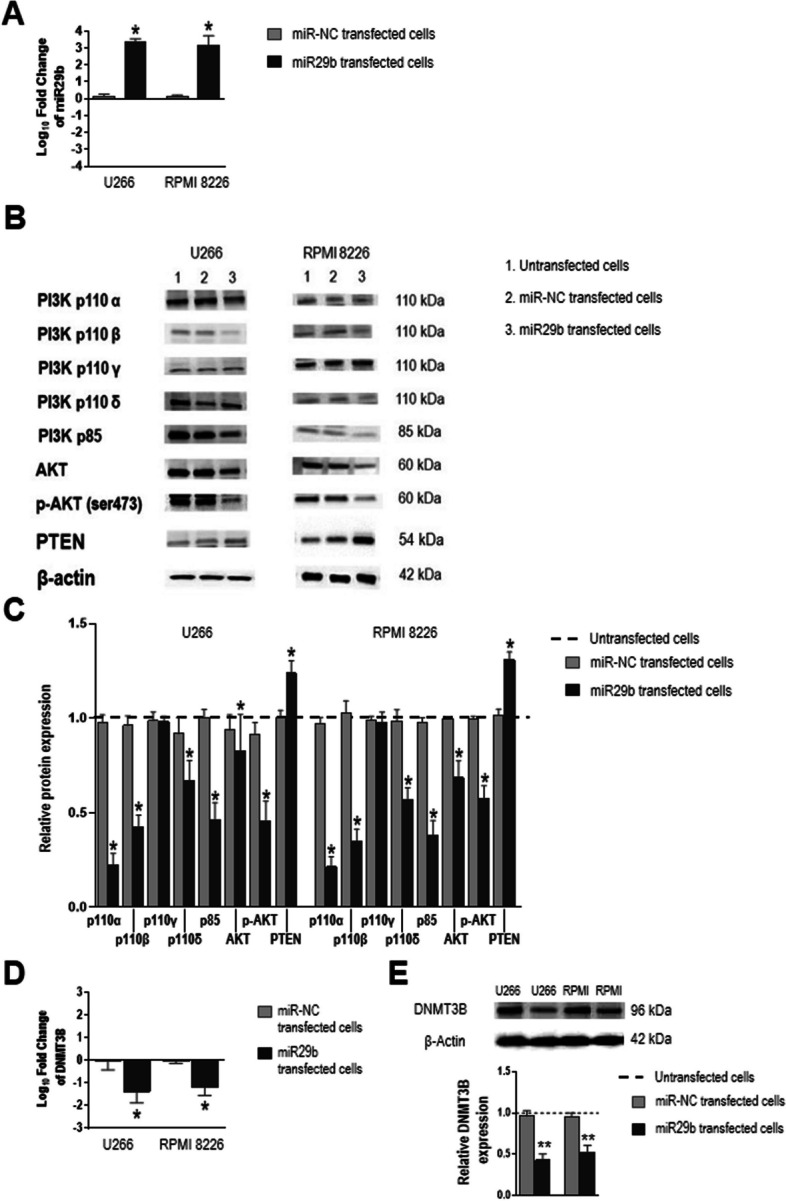
Increased miR29b levels reduce PI3K/AKT pathway components and *DNMT3B* expression. U266 and RPMI 8226 cells were transfected with 10 µM miRNA mimic negative control (miR-NC) or miR29b mimic (miR29b) for 72 h. **A** Levels of miR29b in transfected cells normalized to RNU44 and expressed as fold change relative to the average value for untransfected cells (real-time PCR and 2^−ΔΔCt^ method). **B** Representative western blots of PI3K/AKT pathway components. **C** Densitometric analysis of the experiment shown in (**B**). Band intensities were normalized to β-actin and then to values for untransfected cells (dotted line). **D** Levels of *DNMT3B* mRNA in transfected cells, normalized to *GAPDH* and expressed as fold change relative to the average value for untransfected cells (real-time PCR and 2^−ΔΔCt^ method). **E** Representative western blot and densitometric analysis of DNMT3B in transfected cells. Values were normalized first to β-actin and then to untransfected cells. Values are mean and SD for 7 independent experiments. *P < .05. **P ≤ .01 Wilcoxon signed-rank test

We next investigated whether CD28’s negative regulation of miR29b through the PI3K/AKT pathway is a two-way process, that is whether miR29b expression inhibits the PI3K/AKT pathway. To this end, we transfected U266 and RPMI 8226 cells with a miR29b mimic and achieved a 3-fold increase over endogenous levels (Fig. 3A). Western blotting revealed a marked reduction of PI3Kα, PI3Kβ, and PI3Kδ (*P* = 0.0156), subunit p85 (*P* = 0.0156), AKT (*P* = 0.0469 for U266;* P* = 0.0156 for RPMI 8226) and phospho-AKT (*P* = 0.0156) in transfected cells compared with untransfected cells and cells transfected with miR-NC (Fig. 3B, C). PI3Kγ expression was similar in all treatment conditions (Fig. 3B, C). Transfected cells also displayed significant overexpression of PTEN (*P* = 0.0156), the main negative regulator of the PI3K/AKT pathway (Fig. 3B, C). These results suggest that high levels of miR29b reduce the activity of PI3K/AKT pathway components and, thus, that there exists a regulatory loop between miR29b and the pathway in which they mutually control each other. Moreover, the exogenous addition of miR29b and the consequent reduction of PI3K/AKT signaling decreased the expression of *DNMT3B* mRNA (*P* = 0.0156) and protein (*P* = 0.0078) (Fig. 3D, E), suggesting a relationship among miR29b, PI3K/AKT pathway and DNMT3B.

### The CD28-PI3K-miR29b-DNMT3B axis leads to epigenetic silencing of proteasome subunit genes

**Fig. 4:**
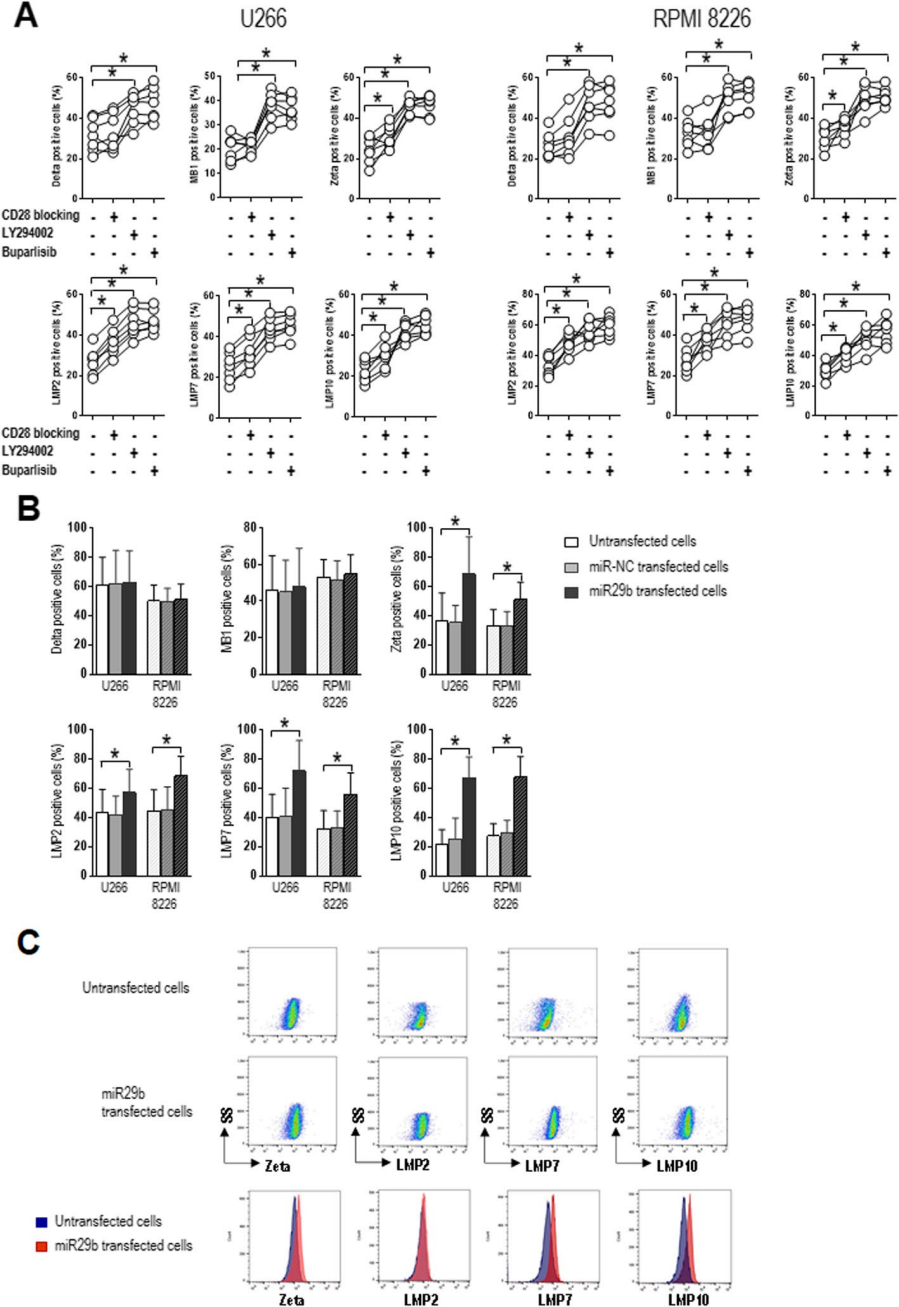
Inhibition of PI3K/AKT signaling and increased miR29b levels restore proteasome subunit expression. U266 and RPMI 8226 cells were stimulated with anti-CD28-loaded particles and (**A**) cultured alone, in the presence of CD28-blocking antibody or the PI3K inhibitor (25 µM LY294002 48 h; 0.5 µM buparlisib 24 h), or (**B**) cultured alone, transfected with 10 µM miRNA mimic negative control (miR-NC) or miR29b mimic (miR29b). Flow cytometry was used to determine the percentage of cells staining positive for each proteasome subunit. Charts show single values (**A**) or mean and SD (**B**) for 7 independent experiments. **C** Representative flow cytometry plots from untransfected and miR29b-transfected cells showing Zeta, LMP2, LMP7 and LMP10 expression. Histogram overlays show the shift in fluorescence. *P < .05. Wilcoxon signed-rank test

Considering the possibility that the expression of proteasome subunits is regulated through promoter DNA methylation by DNMT3B, we then examined proteasome subunit expression in cells treated with the CD28-blocking mAb or the PI3K inhibitors (LY294002 and buparlisib) and in cells transfected with miR29b mimic (Fig. 4). Flow cytometry revealed that CD28 blockade resulted in significantly higher percentages of cells expressing the constitutive proteasome subunit zeta (Z) (*P* = 0.0313) and the three activated immunoproteasome subunits LMP2, LMP7 and LMP10 (*P* = 0.0156 for all) (Fig. 4A). PI3K inhibition increased the percentages of cells expressing all proteasome subunits (*P* = 0.0156 for all) (Fig. 4A). Transfection with miR29b mimic also increased the percentages of cells staining positive for Z, LMP2, LMP7 and LMP10 (*P* = 0.0156 for all) (Fig. 4B, C). These results document the epigenetic regulation of proteasome subunit expression by CD28 along an axis comprising the PI3K/AKT pathway, miR29b and DNMT3B.

**Fig. 5:**
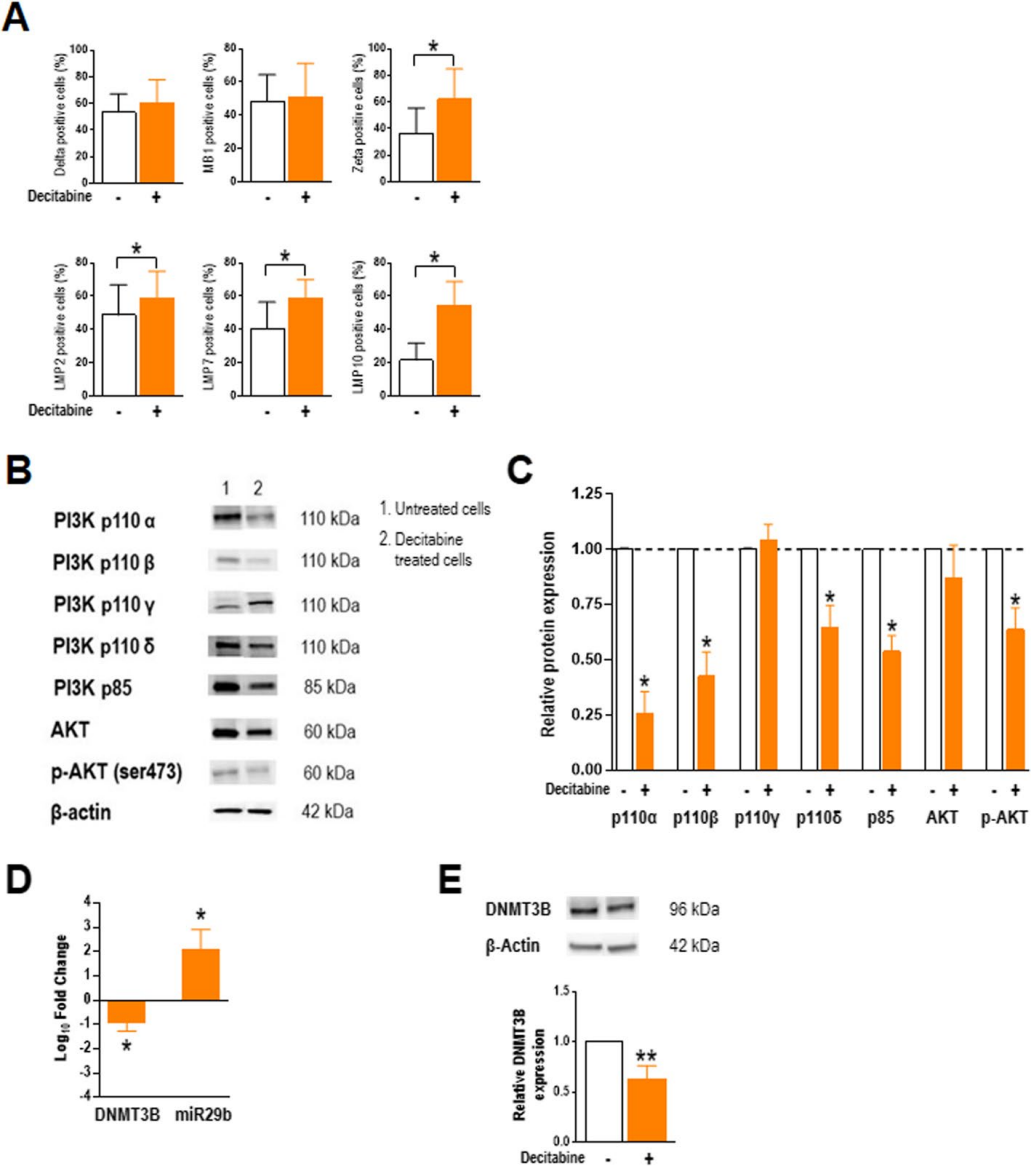
Effects of the DNA methylase inhibitor decitabine. U266 cells were stimulated with anti-CD28-loaded particles and cultured without or with 1 µM decitabine for 72 h. **A** Percentages of cells expressing proteasome subunits, assessed by flow cytometry. **B** Representative western blot of PI3K/AKT pathway components. **C** Densitometric analysis of the experiment shown in (**B**). Band intensities were normalized to β-actin and then to untreated cells (dotted line). **D** Levels of *DNMT3B* mRNA and miR29b normalized to those of *GAPDH* and RNU44, respectively, and expressed as fold change relative to the average value for untreated cells (real-time PCR). **E** Western blot and densitometric analysis of DNMT3B, with values normalized to β-actin and then to untreated cells. Densitometric and fold change values are mean and SD for 7 independent experiments. *P < .05, **P ≤ .01, Wilcoxon signed-rank test

To confirm the epigenetic silencing of plasma cell proteasome subunits, we tested the effects of decitabine, an inhibitor of DNA methylases. Decitabine treatment of U266 cells resulted in increases of cells staining positive for Z (*P* = 0.0313), LMP2 (*P* = 0.0469), LMP7 (*P* = 0.0313) and LMP10 (*P* = 0.0156) (Fig. 5A), similar to the effects of miR29b mimic transfection. We then evaluated the effects of decitabine on the PI3K/AKT pathway and on DNMT3B and miR29b expression. Decitabine treatment of U266 cells significantly decreased protein levels of all PI3K/AKT pathway components except for PI3Kγ (Fig. 5B, C). Decitabine treatment also reduced *DNMT3B* mRNA (*P* = 0.0156) and protein (*P* = 0.0078) and increased miR29b (*P* = 0.0156) levels (Fig. 5D, E). Analogous effects were observed after decitabine treatment of RPMI 8226 cells (data not shown). Again, these results are similar to those obtained with miR29b mimic transfection.

### miR29b enhances the ability of CD8^+^ T cells to recognize and kill myeloma cells

**Fig. 6:**
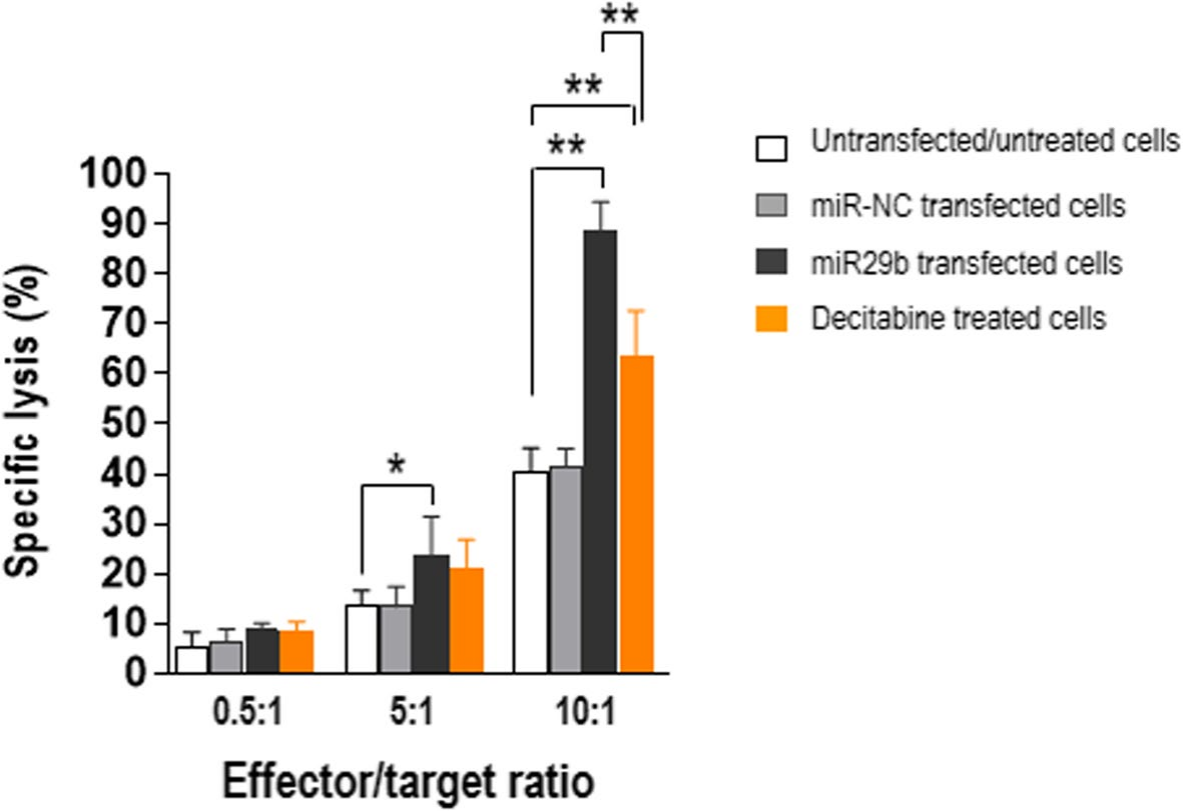
Exogenously added miR29b strongly increases MM plasma cell lysis by bone marrow-derived cytotoxic NY-ESO-1_157–165_-specific CD8^+^ T cells. In this cytotoxicity assay, effectors were CD8^+^ T cells (from bone marrow of 5 MM patients) that had been expanded in vitro in the presence of autologous antigen-presenting cells pulsed with NY-ESO-1_157–165_ peptide. Targets were untransfected, untreated U266 cells or U266 cells transfected with 10 µM miRNA mimic negative control (miR-NC), 10 µM miR29b mimic (miR29b), or treated with 1 µM decitabine for 72 h. *P < .05, ***P ≤ .001. Mann–Whitney U test

We previously found that low levels of proteasome subunits reduced the recognition of MM plasma cells by tumor plasma cell-specific cytotoxic CD8^+^ T lymphocytes [4]. Thus, here we tested whether restoration of high levels of proteasome subunits (by the exogenous addition of miR29b) re-establishes plasma cell killing by CD8^+^ T cells. For cytotoxicity assays, we prepared NY-ESO-1-specific CD8^+^ T cells (from each of the five HLA-A*0201–positive MM patients) to use as effectors. Pentamer staining and flow cytometry revealed that > 95% of the CD8^+^ T cells were specific for the HLA-A*0201–restricted NY-ESO-1_157–165_ epitope (data not shown). As targets, we used U266 cells, which we confirmed by flow cytometry to be HLA-A*0201–positive and NY-ESO-1-expressing (data not shown). NY-ESO-1-specific CD8^+^ T cells recognized and lysed U266 cells transfected with miR29b at effector/target (E/T) ratios of 5:1 (*P* = 0.0397 vs. untransfected cells) and 10:1 (*P* = 0.0079), but not at the lower ratio (Fig. 6). They also lysed decitabine-treated U266 cells at the highest E/T ratio tested (*P* = 0.0079). The lysis of miR-NC-transfected cells was low and similar to that of untransfected cells.

### Levels of miR29b, DNMT3B and proteasome subunit gene methylation predict MM progression

**Fig. 7:**
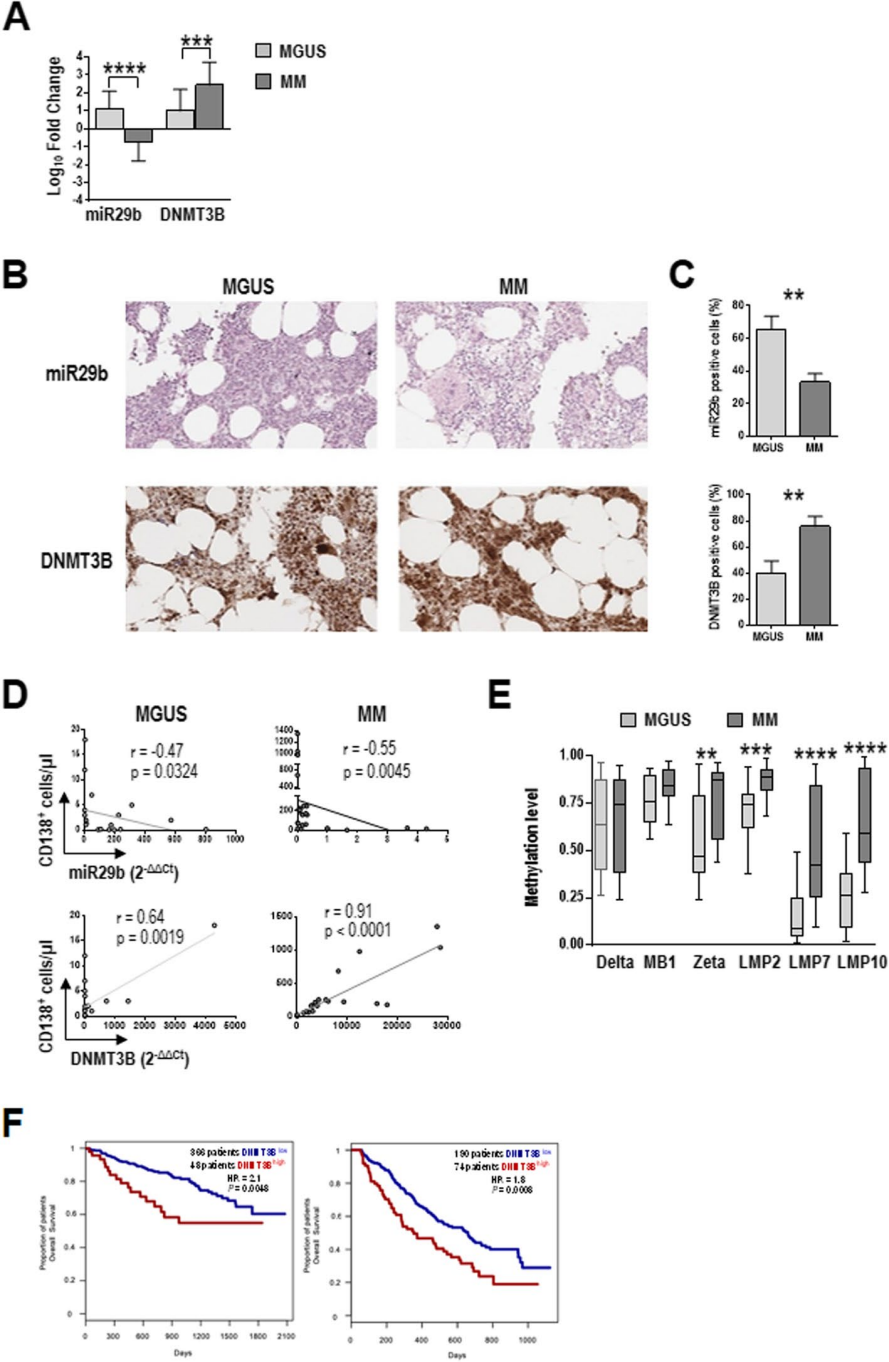
miR29b and DNMT3B expression in plasma cells from MGUS and MM patients. **A** Levels of miR29b and *DNMT3B* mRNA in immunomagnetically purified plasma cells (CD138^+^) from bone marrow of MGUS (*n* = 20) and MM (*n* = 25) patients. Data from real-time PCR and the 2^−ΔΔCt^ method, normalized to RNU44 (miR29b) and *GAPDH* (*DNMT3B*) and expressed as fold change relative to the average value for plasma cells from 10 controls (patients with benign hematological diseases). Values are mean and SD. ***P ≤ .001, ****P < .0001, Mann–Whitney test. **B** Photomicrographs of bone marrow biopsies from MGUS and MM patients stained for miR29b (pink) and DNMT3B (brown). **C** Quantification of positive cells in B. **D** Correlation between numbers of plasma cells (CD138^+^) in bone marrow and levels of miR29b or *DNMT3B* mRNA (2^−ΔΔCt^) in plasma cells from MGUS and MM patients. Spearman’s rank test. **E** Average methylation β values for the promoter regions (TSS1500, TSS200 and 5’UTR) of proteasome subunit genes in plasma cells from MGUS and MM patients. Boxes show median, interquartile range, minimum and maximum. **P ≤ .01, ***P ≤ .001. Mann–Whitney U test. **F** Kaplan–Meier survival curves for MM patients with low or high *DNMT3B* expression in plasma cells. Data from GSE4581 (left) and GSE9782 (right). Log-rank test

Finally, we examined the endogenous expression of miR29b and its target *DNMT3B* in freshly purified plasma cells (CD138^+^) from bone marrow of 20 MGUS and 25 MM patients relative to control patients with benign hematological diseases. Real-time PCR revealed that the relative abundance of miR29b was significantly higher in cells from MGUS than MM patients (*P* < 0.0001), whereas *DNMT3B* mRNA expression was higher in cells from MM than MGUS patients (*P* = 0.0010) (Fig. 7A). Similar findings were observed in bone marrow tissues from these patients: in situ detection of miR29b revealed a higher percentage of stained cells in MGUS than MM samples, while immunohistochemical detection of DNMT3B showed a lower fraction of positive cells in MGUS than MM samples (Fig. 7B, C). Furthermore, miR29b expression levels in plasma cells correlated negatively with the density of plasma cells in bone marrow of both MGUS patients (Spearman’s *r* = -0.47; *P* = 0.0324) and MM patients (*r* = -0.55; *P* = 0.0045) (Fig. 7D). In contrast, *DNMT3B* levels correlated positively with plasma cell number in both MGUS (*r* = 0.64; *P* = 0.0019) and MM (*r* = 0.91; *P* < 0.0001). These results suggest that variations in miR29b and *DNMT3B* levels parallel tumor progression, as revealed by tumor plasma cell burden. Differences between MGUS and MM patients were also seen regarding the extent of DNA methylation in plasma cells: of the six proteasome subunit genes analyzed, lower methylation levels were observed for Z (*P* = 0.0082), LMP2 (*P* = 0.0003), LMP7 (*P* =  < 0.0001) and LMP10 (*P* =  < 0.0001) in MGUS than MM, although the data dispersion tended to be wide (Fig. 7E).

Finally, in a first attempt to evaluate the clinical implications of these molecular dynamics, we used two publicly available datasets from MM patients to investigate the association between *DNMT3B* expression levels and overall survival (data on miR29b expression are not available). Kaplan–Meier analyses revealed that patients with high *DNMT3B* levels had shorter overall survival (Fig. 7F), suggesting that an increase in *DNMT3B* expression may predict poor prognosis.

## Discussion

This study found that stimulation of CD28 triggers the PI3K/AKT pathway in MM plasma cells and downregulates the expression of miR29b. This downregulation results in increased expression of *DNMT3B* and the ensuing methylation and silencing of genes encoding proteasome subunits. Because these subunits are critical for the generation of HLA class I-restricted antigens, such as NY-ESO-1_157–165_, their silencing reduces the recognition of MM plasma cells by tumor-specific cytotoxic CD8^+^ T cells. Compared with plasma cells from MGUS patients, those from MM patients have very low levels of miR29b, high levels of DNMT3B, and more methylation of proteasome subunit gene promoters. These molecular variations parallel the clinical progression from MGUS to MM. Indeed, high levels of DNMT3B associate with poor prognosis in MM patients (according to publicly available datasets).

We also found that the exogenous addition of miR29b to transfected U266 and RPMI 8226 cells reduces PI3K/AKT pathway activity, implying that a regulatory loop exists between miR29b and the PI3K/AKT pathway in which they mutually control each other. This loop regulates DNMT3B expression, given that high exogenous levels of miR29b in myeloma cells also downregulate DNMT3B and restore the expression of proteasome subunits. Similar effects are obtained with decitabine treatment of U266 and RPMI 8226 cells, suggesting that proteasome subunit genes are mainly regulated by methylation. Interestingly, decitabine also enhances miR29b expression in U266 and RPMI 8226 cells, suggesting that low levels of miR29b in MM plasma cells may be due to miR29b promoter methylation (within another self-reinforced epigenetic loop).

Our findings are concordant with results from studies on different tumors. In particular, DNA hypomethylation and restoration of the expression of methylation-silenced genes have been described as results of the forced expression of miR29b in acute myeloid leukemia [20] and lung cancer [21] cells. Methylated CpG sequences in miR-29a/b1 and miR-29b2/c gene promoters have been found in Burkitt lymphoma cells [22]. miR29b has been shown to enhance bortezomib-induced apoptosis in MM plasma cells [11].

There are some caveats associated with our study. Experiments with transfected cells were performed using myeloma cell lines because primary cells recovered from bone marrow were not enough. How miR29b interacts with the PI3K/AKT pathway was not molecularly defined. It might bind directly to the 3'-UTR of the regulatory subunit PI3K p85-β as recently demonstrated in the kidney epithelial cell line NRK-52E [23]. The role of other molecules in influencing the reduction of miR29b could not be ruled out. These molecules may include Myc [24–27], sonic hedgehog [28], nuclear factor kappa-light-chain-enhancer of activated B cells (NF-κB) [28], transforming growth factor beta [29], transcription factor Sp1 [11], and several long non-coding RNAs which act as miR29b sponges [30].

Beyond these limitations, with this paper, we describe a novel pathogenetic mechanism that completes the immunological picture, sketched in our previous reports [4, 5, 31], of what occurs in the bone marrow during MM development. During the MGUS to MM transition, bone marrow becomes enriched in tumor plasma cells, as it is the primary site of their proliferation. It is also enriched in antigen-specific T cells, including CD8^+^ T cells, as it is the preferential site for migration and selective retention of fully reactive cytotoxic lymphocytes [32–34]. In addition, bone marrow increasingly recruits dendritic cells [5]. Dendritic cells, which mainly serve to activate tumor-specific cytotoxic T cells, also interact with MM plasma cells by CD80/86–CD28 binding [5]. This binding activates, in plasma cells, the PI3K/AKT pathway, which downregulates miR29b expression and consequently upregulates *DNMT3B*. High levels of this methyltransferase result in the epigenetic silencing of genes that encode immunoproteasome subunits. These subunits are involved in the processing of tumor antigens and are essential for the generation of HLA class I tumor antigen-derived peptides which are presented to CD8^+^ T cells. As a result, MM plasma cells are not recognized by tumor-specific cytotoxic CD8^+^ T cells and thus are not killed by them. The more dendritic cells accumulate in the bone marrow, the more MM plasma cells become less immunogenic and evade CD8^+^ T cell surveillance.

In this picture, miR29b and DNMT3B emerge as important molecules with interesting clinical potential. They may be predictive biomarkers for identifying MGUS patients who are more likely to develop MM or MM patients with poor prognosis. At the same time, they may open new avenues for miR29b-based epi-therapeutic strategies for MM.

### Supplementary Information

**Supplementary Material 1.**

**Supplementary Material 2.**

## Data Availability

All data generated in this study are available within the article, its supplemental information, and from the corresponding author upon reasonable request.

## Abbreviations

MM: Multiple Myeloma
MGUS: Monoclonal gammopathy of undetermined significance
HLA: Human leukocyte antigen
PI3K: Phosphatidylinositol-3-kinase
DNMTs: DNA methyltransferases
ATCC: American Type Culture Collection
FBS: Fetal bovine serum
BMMC: Bone marrow mononuclear cells
mAb: Monoclonal antibodies
GAPDH: Glyceraldehyde-3-phosphate dehydrogenase
β2m: Beta2-microglobulin
TBP: TATA-box binding protein
miR29b: MiR29b mimic
miR-NC: MiRNA mimic negative control
LDH: Lactate dehydrogenase
ChAMP: Chip Analysis Methylation Pipeline
TSS1500: 200–1500 Bp upstream of the transcription start site
TSS200: 0–200 Bp upstream of the transcription start site
UTR: Untranslated region
PTEN: Phosphatase and tensin homolog
