# Supplementary material for: Immune escape of multiple myeloma cells results from low miR29b and the ensuing epigenetic silencing of proteasome genes
Source: Biomark Res. 2024 Apr 23;12:43. doi: 10.1186/s40364-024-00592-y (PMC11040965; doi:10.1186/s40364-024-00592-y)
Supplement: Supplementary file 1 — Supplementary Material 1. [file 40364_2024_592_MOESM1_ESM.docx]

**Supplemental Table 1. Antibodies used in the study.**

| **Assay** | **Antigen** | **Host**  **species** | **Conjugation** | **Dilution** | **Source (ID)** | **RRID** |
| --- | --- | --- | --- | --- | --- | --- |
| **Cell**  **culture** | CD28 | Mouse | -- | -- | Thermo Fisher Scientific  (#16-0288-81) | AB_468924 |
|  |  |  |  |  |  |  |
| **Western**  **blotting** | PI3K p85 | Rabbit | -- | 1:1000 | Cell Signaling (#4257) | AB_659889 |
|  | PI3K p110α | Rabbit | -- | 1:500 | Cell Signaling (#4249) | AB_2165248 |
|  | PI3K p110β | Rabbit | -- | 1:500 | Cell Signaling (#3011) | AB_2165246 |
|  | PI3K p110γ | Rabbit | -- | 1:500 | Cell Signaling (#5405) | AB_1904087 |
|  | PI3K p110δ | Rabbit | -- | 1:500 | Cell Signaling (#34050) | AB_2799043 |
|  | AKT^a^ | Rabbit | -- | 1:1000 | Cell Signaling (#9272) | AB_329827 |
|  | phospho-AKT (Ser473) | Rabbit | -- | 1:2000 | Cell Signaling (#4060) | AB_2315049 |
|  | DNMT3B | Rabbit | -- | 1:500 | Cell Signaling (#67259) | AB_2799723 |
|  | PTEN^a^ | Rabbit | -- | 1:1000 | Cell Signaling (#9552) | AB_10694066 |
|  | β-Actin | Mouse | -- | 1:5000 | Thermo Fisher Scientific  (#MA1-140) | AB_2536844 |
|  | Mouse IgG^a^ | Goat | HRP | 1:3000 | Bio-Rad (#170-6516) | AB_2921252 |
|  | Rabbit IgG | Goat | HRP | 1:3000 | Bio-Rad (#170-6515) | AB_11125142 |
| **Flow**  **cytometry** | CD28 | Mouse | PE | -- | Miltenyi Biotec (#130-123-782) | AB_2802058 |
|  | CD138 | Mouse | PE | -- | Beckman Coulter (#A54190) | NA |
|  | CD8 | Mouse | FITC | -- | Beckman Coulter (#A07756) | NA |
|  | NY-ESO-1 | Mouse | PE | -- | Proimmune | NA |
|  | Proteasome  subunit Delta | Mouse | FITC | -- | Massachusetts General Hospital, Harvard Medical School | NA |
|  | Proteasome  subunit MB1 | Mouse | FITC | -- | Massachusetts General Hospital, Harvard Medical School | NA |
|  | Proteasome  subunit Zeta | Mouse | FITC | -- | Massachusetts General Hospital, Harvard Medical School | NA |
|  | Proteasome  subunit LMP2 | Mouse | FITC | -- | Massachusetts General Hospital, Harvard Medical School | NA |
|  | Proteasome  subunit LMP7 | Mouse | FITC | -- | Massachusetts General Hospital, Harvard Medical School | NA |
|  | Proteasome  subunit LMP10 | Mouse | FITC | -- | Massachusetts General Hospital, Harvard Medical School | NA |
| **Immunohistochemistry** | |  |  |  |  |  |
| DNMT3B antibody^a^ | | Rabbit | -- | 1:250 | Abcam (#ab2851) | AB_303356 |

Antibodies are monoclonal unless otherwise indicated. DNMT3B, DNA methyltransferase 3B; FITC, fluorescein isothiocyanate; HRP, horseradish peroxidase; NA, not available; PE, phycoerythrin; PI3K, phosphatidylinositol 3-kinase.

^a^ Polyclonal.
