# Supplementary figures and images for: Immune escape of multiple myeloma cells results from low miR29b and the ensuing epigenetic silencing of proteasome genes
Source: Biomark Res. 2024 Apr 23;12:43. doi: 10.1186/s40364-024-00592-y (PMC11040965; doi:10.1186/s40364-024-00592-y)

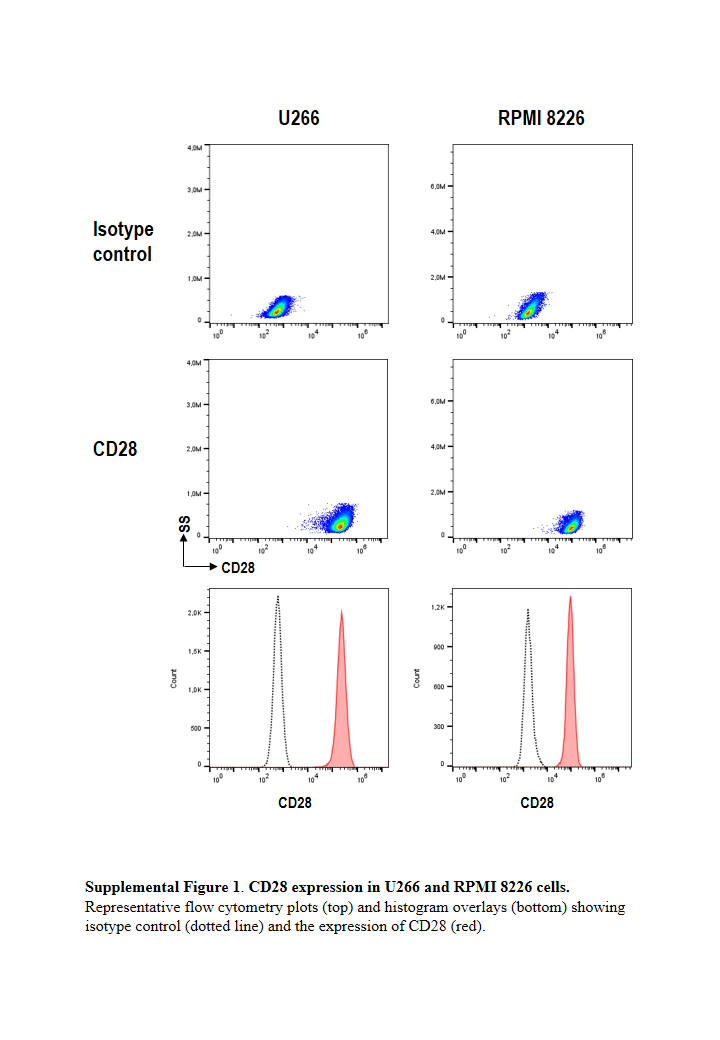

Supplement: Supplementary file 2 — Supplementary Material 2. [file 40364_2024_592_MOESM2_ESM.tif]
